# Magnoflorine Attenuates Cerebral Ischemia-Induced Neuronal Injury via Autophagy/Sirt1/AMPK Signaling Pathway

**DOI:** 10.1155/2022/2131561

**Published:** 2022-09-10

**Authors:** Hai Liang, Xin Chang, Runan Xia, Wei Wu, Hongju Guo, Miao Yang

**Affiliations:** ^1^Department of Pharmacy, The People's Hospital of Bozhou, Bozhou, Anhui, China; ^2^Department of Neurobiology, Beijing Institute of Basic Medical Sciences, Beijing, China; ^3^Department of Pharmacy, Strategic Support Force Medical Center, Beijing, China; ^4^Department of Neurology, The People's Hospital of Bozhou, Bozhou, Anhui, China

## Abstract

Ischemic stroke is a common cause of permanent disability worldwide. Magnoflorine has been discovered to have good antioxidation, immune regulation, and cardiovascular system protection functions. However, whether magnoflorine treatment protects against cerebral ischemic stroke and the mechanism of such protection remains unknown. Here, we investigated the effect of magnoflorine on the development of ischemic stroke disorder in rats. A middle cerebral artery occlusion (MCAO) model followed by 24 h reperfusion after 90 min ischemia was used. The rats were treated with magnoflorine (10 mg/kg or 20 mg/kg) for 15 consecutive days. The neurological deficit scores, cerebral infarct volume, and brain water content were measured. The neuronal density was determined using Nissl and NeuN staining. The oxidative stress levels were determined using commercial kits. Immunofluorescence staining of LC3 and western blot assay for LC3 and p62 were used to assess autophagy. Magnoflorine treatment significantly reduced the cerebral infarct volume and brain water content and improved the neurological deficit scores in the rat MCAO model. In addition, magnoflorine ameliorated neuronal injury and neuron density in the cortex of rats. Magnoflorine also prevented oxidative damage following ischemia, reflected by the decrement of nitric oxide and malondialdehyde and the increase of glutathione (GSH) and GSH peroxidase. Moreover, the fluorescence intensity of LC3 and the ratio of LC3-II to LC3-I were remarkably downregulated in ischemic rat administration of magnoflorine. Finally, the expression levels of p62, sirtuin 1 (Sirt1), and phosphorylated-adenosine monophosphate-activated protein kinase (AMPK) were upregulated with magnoflorine. Magnoflorine attenuated the cerebral ischemia-induced neuronal damage, which was possibly associated with antioxidative stress, suppression of autophagy, and activation of the Sirt1/AMPK pathway in the rats.

## 1. Introduction

Stroke has become a severe issue worldwide, as it is the second leading cause of death and a principal factor for disability. It affects around 13.7 million people and kills roughly 5.5 million annually [1]. Ischemic stroke, the most common type of stroke, is a neurological disease characterized by blockage of the cerebral vessels. Ischemic occlusion results in thrombotic and embolic conditions in the brain that cause serious stress and untimely neurocyte death [2]. Although nanocarriers present an effective diagnosis or treatment for ischemic stroke [3], novel agents and therapeutic strategies for stroke are still needed.

Several natural alkaloid compounds, such as berberine, have shown beneficial effects on the basic treatment of stroke [4]. Magnoflorine is an alkaloid product derived from Ziziphi spinosae semen (ZSS), the dried and ripe seed of Ziziphus jujuba [5]. ZSS, as a popular herb that has a long history in China, has been documented to protect the liver functions, quiet the heart, and arrest sweating traditional Chinese medicine [5]. However, scientific studies on the biological activity of magnoflorine are still insufficient. Until now, pharmacological studies have reported that magnoflorine exhibits an antioxidation effect [6], regulating immune function [7], and protective efficacy on the cardiovascular system [8]. Moreover, mice treated with magnoflorine combining berberine can block long-term memory impairment in a passive avoidance test [9]. Though the central activity of magnoflorine has been confirmed, its neuroprotective properties toward the brain have not yet been fully elucidated.

Some key events contributing to worsening stroke progression are energy failure, oxidative stress, inflammation, excessive autophagy, impairment of the blood–brain barrier, and activation of microglial cells [10]. Among these factors, excessive autophagy has been identified as a crucial signal that results in accelerating the severity of stroke [11, 12]. Tao et al. have reported that autophagosome is observed in the brain tissues with middle cerebral artery occlusion [11]. Inhibition of autophagy-associated neuro death conferred the neuroprotective action both *in vivo* and *in vitro* [12]. In addition, it has been widely accepted that oxidative stress triggers mitochondrial dysfunction and neuronal death which causes irreversibly neurological sequelae [13]. An essential finding has been shown that the oxidative markers including inducible nitric oxide synthase (iNOS), NADPH oxidase-1 (NOX-1), and NOX-2, are notably elevated in the ischemic stroke mice model [14]. Clinical researches have also demonstrated a close correlation between increased oxidative stress and cerebral ischemia [15]. Therefore, reducing levels of autophagy and oxidative stress may be a potential therapeutic strategy against stroke insult.

Sirtuin 1 (Sirt1) is the highest homologous gene in seven Sirtuin 2 genes. Small molecule modulators of sirtuins have been reported to possess beneficial effects on the treatment of cardiovascular and neurodegenerative disorders [16, 17]. Among them, Sirt1 exhibits a crucial effect on energy metabolism, cell survival, and aging through Sirt1/adenosine monophosphate-activated protein kinase (AMPK) signaling pathway [18]. There is evidence that the protective effects of Sirt1 are closely associated with the activation of autophagy. Notably, magnoflorine can efficiently downregulate the protein expression of LC3B in type 2 diabetic rat suggesting an inhibition effect of magnoflorine on autophagy activation [19].

In this study, we aimed to assess the neuroprotective effects and underlying mechanisms of magnoflorine on ischemic stroke in a middle cerebral artery occlusion (MCAO) rat model.

## 2. Materials and Methods

### 2.1. Animals

Male Sprague-Dawley rats (220 ± 10 g) were purchased from the Laboratory Animal Center of Zhejiang Academy of Medical Sciences (Hangzhou, China). All the animals were maintained in an environmentally controlled room (22–25°C, 12 h light–dark cycle, relative humidity 55%) and had free access to food and water. The rats received humane care in accordance with the National Institutes of Health Guide for the Care and Use of Laboratory Animals. The animal experiments were approved by the Ethics Committee of Laboratory Animal Care and Welfare, Zhejiang Academy of Medical Sciences (number 2019–193).

### 2.2. Experimental Protocols

**Figure 1:**
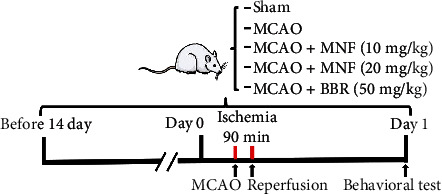
Schematic of the experimental protocols. Rats were subjected to cerebral ischemia for 90 min and reperfusion for 24 h MNF (10 mg/kg/day, 20 mg/kg/day, intragastric) or BBR (50 mg/kg/day, intragastric) was administered once a day for consecutive 14 days before MCAO, and 1 day after surgery. Behavioral tests were performed on day 1 following cerebral ischemia. MNF, magnoflorine; BBR, berberine; MCAO, middle cerebral artery occlusion.

The rats were randomly divided into five groups (with 18 rats for each group): the sham group, MCAO group, MCAO + magnoflorine 10 mg/kg group,MCAO + magnoflorine 20 mg/kg group, and MCAO + berberine 50 mg/kg group. The rats in the MCAO models were subjected to brain ischemia for 90 min and reperfusion for 24 h. Magnoflorine and berberine (intragastric; Sigma-Aldrich, MO, USA) were dissolved with dimethyl sulfoxide (DMSO) as a stock solution and diluted with saline (DMSO final concentration <0.5%). The sham and MCAO groups received equal volumes of saline with the same amount of DMSO. The described treatment groups were administered agents once a day for 14 days before MCAO and 1 day after surgery. The dose of magnoflorine was chosen according to a previous study with minor modifications [20]. Behavioral tests were performed on day 1 following MCAO (Figure 1). All the rats were anesthetized with phenobarbital sodium (40 mg/kg, *i.p.*), and blood was collected from the aorta abdominalis. The rats were then euthanized by decapitating, and brain tissues were quickly obtained for subsequent experiments.

### 2.3. Establishment of the MCAO Model

The MCAO procedure was performed as previously described [21]. In brief, the rats were fasted for 10 h and then anesthetized with phenobarbital sodium (40 mg/kg, *i.p.*). Subsequently, a narrow incision was made along the midline of the neck, and the right common carotid artery, external carotid artery (ECA), and internal carotid artery were carefully exposed and separated. The ECA was then cut obliquely, and a 3–0 nylon suture coated with silicone (2636, Xinong Bioengineering Institute, Beijing, China) was inserted until slight resistance was felt, effectively blocking the middle cerebral artery. The suture was withdrawn after 90 min occlusion to achieve reperfusion. The rats in the sham group underwent the same procedure, except for filament insertion. Following surgery, the rats were housed in a 25°C box heated with lamps for 2 h.

### 2.4. Behavioral Testing

At 24 h after MCAO, the neurological function of the rats was assessed using the modified neurological severity score (mNSS) [22], which could reflect muscle status, vision, touch, abnormal movement, proprioception, and reflex systems. A score of 0 indicates no neurologic deficit; 1–6, mild injury; 7–12, moderate injury; and 13–18, severe injury. The higher the score, the more severe the neurological damage. Behavioral testing was performed by an investigator blinded to the experiments.

Longa neurological examination was applied to assess the neurological deficit. The Longa behavior test was graded on a scale of 0–5 points: 0, no neurological deficit; 1, failure to fully extend the left forelimb, mild focal neurological deficit; 2, circling to the left, moderate neurological deficit; 3, falling to the left, severe focal deficit; 4, no spontaneous walking and depressed level of consciousness; and 5, death. The Longa behavior test was performed by a practiced investigator who was blinded to the experiments.

### 2.5. Assignment of Brain Water Content

After the evaluation of the behavioral test results, the brain tissues were immediately collected from all the groups and weighted (wet weight). The samples were then again after dehydration in an oven at 100°C for 24 h (dry weight). The brain water content was calculated using the following formula% = (wet weight − dry weight)/wet weight × 100.

### 2.6. Evaluation of Cerebral Infarct Volume

The cerebral infarct volume was measured with 2, 3, 5-triphenyltetrazolium chloride (TTC) staining, as before described [23]. The brain tissues were cut into six slices and stained with 2% TTC at 37°C for 20 min. The samples were subsequently fixed in 4% paraformaldehyde for 6 h. The infarct size and total hemispheric areas were determined using the ImageJ analysis software (National Institutes of Health, Bethesda, USA). The percentage of cerebral ischemic volume was calculated.

### 2.7. Nissl Staining

The rats were anesthetized with phenobarbital sodium (40 mg/kg, *i.p.*) and perfused with saline followed by 4% paraformaldehyde for fixation. The brain tissues were embedded in paraffin and cut into 5-*μ*m-thick slides. The samples were deparaffinized using xylene and dehydrated in gradient concentrations of ethanol. After being stained with a 0.2% Nissl staining solution, the slides were observed with a light microscope (Leica Microsystems, Wetzlar, Germany). The number of injured neurons was determined using the ImageJ software.

### 2.8. Immunofluorescence Analysis

The brain sections in all the groups were incubated with 10% donkey serum for 1 h at room temperature. The sections were subsequently treated with the primary antibodies, including NeuN (Abcam, Cambridge, UK) or LC3 (Cell Signaling Technology, Danvers, USA) at 4°C overnight. After being washed with phosphate-buffered saline (PBS) 3 times, the samples were incubated with Alexa Fluor 488-conjugated IgG secondary antibodies for 2 h at room temperature. The slides were further incubated with DAPI (Beyotime Institute of Biotechnology, Shanghai, China) for 15 min at room temperature. Images were taken under a Leica microscope.

### 2.9. Measurement of Nitric Oxide (NO), Malondialdehyde (MDA), Glutathione (GSH), and GSH Peroxidase (GSH-PX)

Blood plasma was collected and centrifuged at 1,500 g for 15 min at 4°C. The supernatant was used for NO measurement with a commercial kit (Nanjing Jiancheng Bioengineering Institute, Nanjing, China). The contents of MDA, GSH, and GSH-PX in brain tissues were assessed using kits (Nanjing Jiancheng Bioengineering Institute). The protocols were performed according to the manufacturer's instructions.

### 2.10. Western Blot Analysis

Protein in the ischemic cortex tissue was extracted with a commercial protein extraction kit (Beyotime), and the protein quantity was measured with a BCA protein assay kit (Beyotime), following the manufacturer's protocols.

A total of 40 *μ*g protein samples were separated through sodium dodecyl sulphate-polyacrylamide gel electrophoresis (10–15%), and the protein was transferred to nitrocellulose membranes. After the samples were blocked in 10% skimmed milk for 1 h at room temperature, they were incubated with primary antibodies against LC3 (1 : 1000; Sigma-Aldrich), p62 (1 : 1000; Abcam), p-AMPK (1 : 1000; Cell Signaling Technology, MA, USA), AMPK (1 : 1000; CST), Sirt1 (1 : 1000; CST), and glyceraldehyde 3-phosphate dehydrogenase (GAPDH; 1 : 3,000; Bioworld Technology, St Louis Park, USA) at 4°C overnight. The membranes were washed with PBS 3 times, for 5 min each time, and incubated with horseradish peroxidase-conjugated IgG (1 : 5,000, Cell Signaling Technology) secondary antibodies for 2 h at room temperature. Specific protein signals were visualized with an ECL Western Blotting Detection System (Millipore, Billerica, MA, USA). The bands were analyzed by ImageJ software and normalized for GAPDH expression.

### 2.11. Statistical Analysis

All the data are presented as mean ± standard deviation (SD). Statistical significance (*P* <  0.05) was analyzed using a one-way analysis of variance (ANOVA) followed by the Newman–Keuls multiple comparison test. Data analyses were performed with GraphPad software (Prism Version 8.01).

## 3. Results

### 3.1. Magnoflorine Ameliorated Cerebral Ischemic Injury in Rats

**Figure 2:**
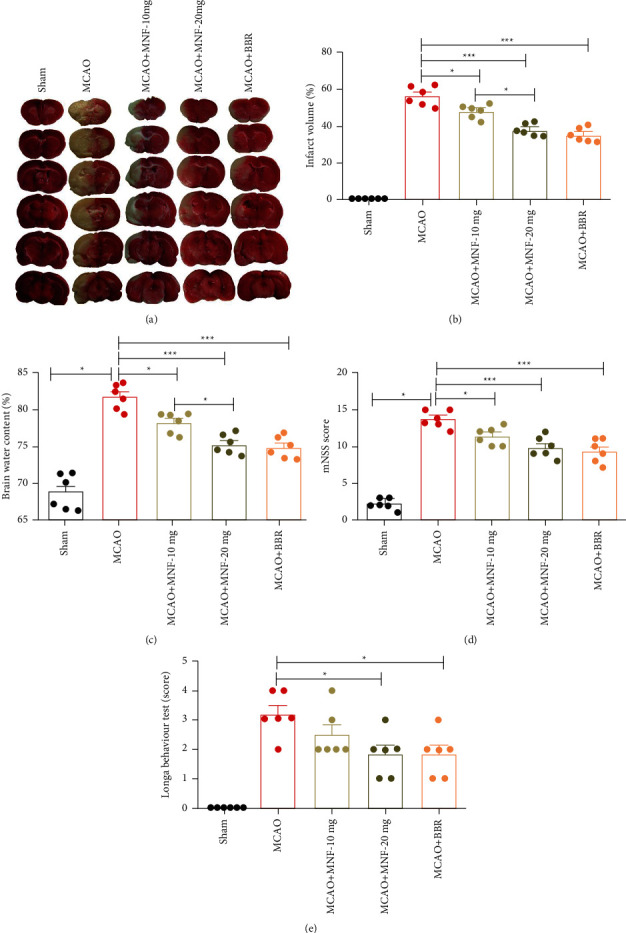
Effect of magnoflorine on infarct volume, brain water content, and neurological function following cerebral ischemia in rats. The representative images of TTC staining of brain sections (a) and quantification of the infarct volume (b) were shown. Brain water content (c), mNSS score (d), and longa behavior test score (e) were measured after cerebral ischemia. Values are mean ± SEM, *n* = 6 per group. ^*∗*^*P* < 0.05, ^*∗∗∗*^*P* < 0.001. MNF, magnoflorine; BBR, berberine; MCAO, middle cerebral artery occlusion.

To investigate the protective effects of magnoflorine against cerebral ischemic injury, the cerebral infarct volume, brain water content, mNSS values, and Longa behavior test scores were examined in rat models. The rats were subjected to 90 min MCAO and 24 h reperfusion. As shown in Figures 2(a) and 2(b), magnoflorine at both 10 mg/kg and 20 mg/kg significantly reduced the infarct volume compared with the MCAO group (*P* <  0.05). Similarly, the percentage of brain water content was significantly lessened in the magnoflorine treatment groups compared to the MCAO group (Figure 2(c), *P* <  0.05). The MCAO rats exhibited highly mNSS scores and Longa behavior test scores, whereas magnoflorine administration (20 mg/kg) remarkably decreased both neurological deficit scores (Figures 2(d), 2(e), *P* <  0.05). Magnoflorine at 10 mg/kg significantly improved the mNSS scores (Figure 2(d), *P* <  0.05) and slightly reduced the Longa behavior test scores (Figure 2(e), *P* <  0.05). In previous studies, berberine decrease the infarct volume and ameliorated the neurological deficit scores in a cerebral ischemia-reperfusion-induced injury model [4]. Thus, berberine administration was used as a positive control in the current study. Berberine displayed neuroprotective effects similar to those displayed by magnoflorine (20 mg/kg). Taken together, these data suggest that magnoflorine has protective effects on cerebral MCAO-induced injury in rats.

### 3.2. Magnoflorine Prevented Neuronal Damage in the Cortex following Cerebral Ischemia in Rats

**Figure 3:**
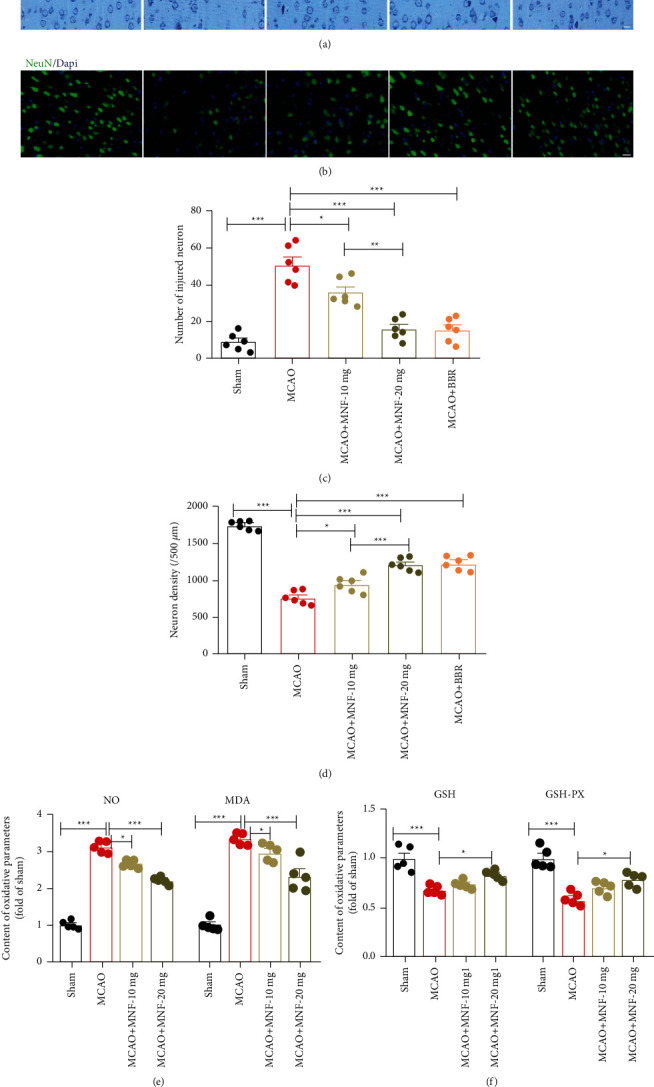
Effect of magnoflorine on neuronal damage and oxidant stress following cerebral ischemia in rats. Representative images of the Nissl staining in the cerebral cortex (a) and quantification the injured neurons (c) were shown. Brain sections were stained with NeuN (green) and DAPI (blue) to monitor the density of neurons. (b) Representative immunofluorescent staining in the cerebral cortex of rats. (d) The neuron density was quantified. (e-f) The concentrations of NO, MDA, GSH, GSH-PX in the serum or brain tissues were detected. Scale bar, 100 µm. Values are mean ± SEM, *n* = 5 per group. ^*∗*^*P* < 0.05, ^*∗∗*^*P* < 0.01, ^*∗∗∗*^*P* < 0.001. MNF, magnoflorine; BBR, berberine; MCAO, middle cerebral artery occlusion; NO, nitric oxide; MDA, malondialdehyde; GSH, glutathione; PX, peroxidase.

To explore the influence of magnoflorine on neuronal damage in the cortex of MCAO rats, Nissl staining and NeuN staining were used the results showed marked neuronal changes, for example, the cell bodies were shrunken and deeply stained and the Nissl body disappeared in the MCAO group (Figures 3(a) and 3(c), *P* <  0.05). The density of the NeuN-positive cells in the cortex, corresponding to the number of neurons, was obviously smaller in the MCAO group than in the sham group (Figures 3(b) and 3(d), *P* <  0.05). However, magnoflorine treatment greatly reduced the injured neurons and elevated the neuron density at both the 10 mg/kg and 20 mg/kg doses compared with the MCAO group (Figures 3(a)–3(d), *P* <  0.05). Berberine treatment showed similar effects to magnoflorine. The results indicate that magnoflorine alleviates neuronal damage in the cortex following cerebral ischemia in rats.

### 3.3. Magnoflorine Inhibited Oxidant Stress following Cerebral Ischemia in Rats

To clarify the role of magnoflorine in antioxidant stress in cerebral MCAO-induced injury, the oxidative parameters were measured in the rats. The serum NO and MDA in the cerebral tissues were significantly elevated in the MCAO group compared to the sham group, while magnoflorine at both doses remarkedly reduced the levels of NO and MDA in the rats following cerebral ischemia (Figure 3(e), *P* <  0.05). In addition, the antioxidative parameters of GSH and GSH-PX were lower in the MCAO group than in the sham group. Magnoflorine at 10 mg/kg and 20 mg/kg was sufficient to increase the GSH and GSH-PX contents compared with the MCAO group (Figure 3(f), *P* <  0.05). These data suggest that magnoflorine plays a role in relation to antioxidant stress in the cerebral ischemic model.

### 3.4. Magnoflorine Mediated Autophagy/Sirt1/AMPK Pathway following Cerebral Ischemia in Rats

**Figure 4:**
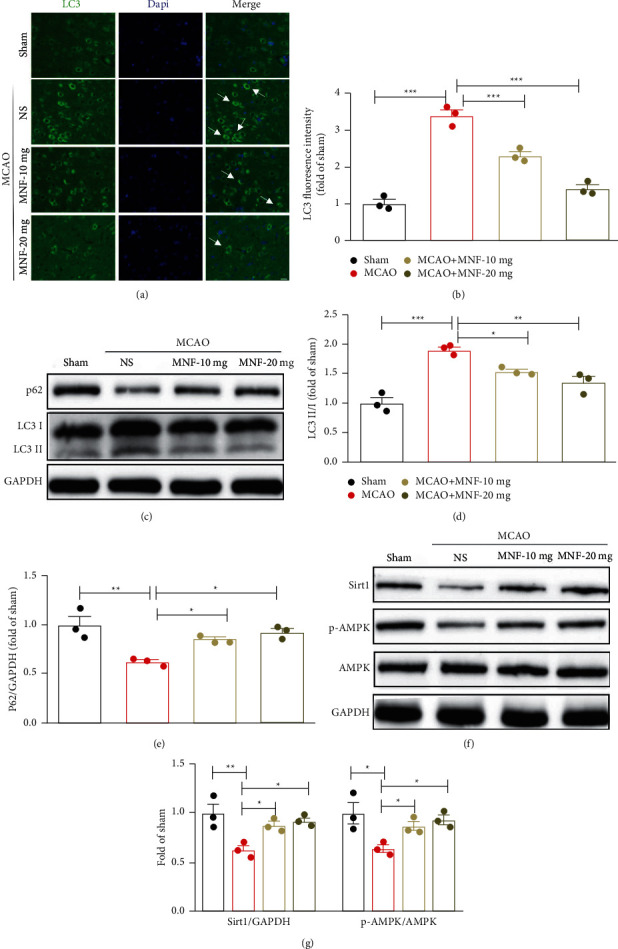
Effect of magnoflorine on autophagy/Sirt1/AMPK pathway following cerebral ischemia in rats. Brain sections were stained with LC3 (green) and DAPI (blue) to reveal the expression of LC3 in the cortex. Representative images of the LC3 staining (a) and quantification the fluorescence intensity of LC3 (b) were shown. White arrows indicate LC3-positive cells. (c) Protein levels of LC3 II to LC3 I and p62 were analyzed using a western blot. (d–e) The bar graphs. (f–g) Sirt-1 and p-AMPK expressions in brain tissues, and the statistical data. Scale bar, 100 *µ*m. Values are mean ± SEM, n = 3 per group. ^*∗*^*P* < 0.05, ^*∗∗*^*P* < 0.01, ^*∗∗∗*^*P* < 0.001. MNF, magnoflorine; MCAO, middle cerebral artery occlusion.

To elucidate the molecular mechanisms of magnoflorine on the neuroprotective effects in the rat MCAO model, the level of autophagy, and the expressions of Sirt1 and phosphorylated-AMPK (p-AMPK) were evaluated in the rats. Stronger and more extensive staining for LC3 appeared in the cortexes of the rats in the MCAO group than in the sham group (Figures 4(a) and 4(b), *P* < 0.001). Importantly, the ratio of LC3-II to LC3-I was greatly upregulated, and the expression of the p62 protein was downregulated in the MCAO group compared to the sham group (Figures 4(c) and 4(e), *P* <  0.05). By contrast, reductions in fluorescence intensity and in the protein expression of LC3-II to LC3-I were detected in the brain tissues from the magnoflorine treatment groups (Figure 4(a) and 4(d), *P* <  0.05). In addition, magnoflorine treatment at both doses significantly decreased the level of p62 (Figure 4(e), *P* <  0.05). It also elevated the decreased expression levels of Sirt1 and p-AMPK in a dose-dependent manner in the MCAO group (Figures 4(f) and 4(g), *P* <  0.05). The results demonstrate that the neuroprotective effect of magnoflorine may be related to the autophagy/Sirt1/AMPK pathway in ischemic rats.

## 4. Discussion

Through the current study, we provided evidence that magnoflorine treatment decreased the cerebral infarct volume and brain edema, improved neurological deficits, and attenuated the neuronal damage in rat MCAO models. More importantly, magnoflorine significantly suppressed oxidative stress as reflected by the decreases in NO and MDA and the increases in GSH and GSH-PX. We further explored whether magnoflorine mediated the autophagy/Sirt1/AMPK pathway following ischemia in rats. The results indicate that the protective effects of magnoflorine on cerebral ischemic stroke are related to the suppression of oxidative stress and the mediating autophagy/Sirt1/AMPK pathway in rats.

It is well known that tissue plasminogen activator (tPA) is the only Food and Drug Administration-approved agent for the treatment of ischemic stroke. However, a patient with ischemic stroke must receive this treatment within a 4.5 h golden-time window. Less than 10% of the patients have successfully received tPA treatment in the clinic [24]. New therapeutic strategies for the improvement of ischemia reperfusion injury in the brain must be developed.

Magnoflorine is an important alkaloid that has received increasing attention because of its numerous pharmacological activities, such as antioxidant, anti-inflammation, anti-diabetes, neuropsychopharmacology, hypotension, and antifungal effects [19, 25, 26]. It has been reported to inhibit lipopolysaccharide-induced apoptosis in murine macrophage cells [27]. Su et al. found that magnoflorine negatively regulates the BRAF protein in the *Pseudomonas aeruginosa* PAK strain involved in the mitogen-activated protein kinase (MAPK) signaling pathway and that it was a potential NF-*κ*b inhibitor [28]. Importantly, magnoflorine is prone to penetrate the blood–brain barrier and has shown marked central activity in mice [9]. Administration of magnoflorine has also been reported to improve the short-and long-term acquisition of memory and learning [20]. In this study, we used Sprague-Dawley rats subjected to MCAO surgery as an experimental model of ischemic stroke. We found that treatment with different doses of magnoflorine (10 mg/kg/day and 20 mg/kg/day) for 15 consecutive days significantly decreased the infarct volume, brain water content, and neurological deficit scores. In addition, magnoflorine obviously reduced the number of injured neurons in the cortexes of the rats. These results suggest that magnoflorine may attenuate MCAO-induced neuronal injury in rats. However, the mechanisms underlying ischemic stroke have not yet been fully understood.

One generally held view is that oxidative stress contributes to cerebral injury in stroke and reperfusion after stroke [29]. The term oxidative stress' regard is a condition in which cells receive excessive molecular oxygen or its chemical derivatives. NO and MDA have profound effects on stroke pathogenesis, resulting from the high susceptibility of the cerebra to reactive oxygen species-induced injury [29]. Overwhelming production of oxidative stress leads to autophagy induction in ischemic stroke. Lu et al. reported that autophagosome formation is increased in neuronal cells subjected to oxygen-glucose deprivation (OGD), corresponding with upregulated mRNA and protein levels of LC3 [30]. These results suggest a close association between oxidative stress and autophagy in brain ischemic damage. Nevertheless, whether autophagy is a friend or an opponent still remains controversial. Both in vivo and in vitro studies have shown that the administration of 3-methyladenine (3-MA), a selective autophagy inhibitor, largely protects neurons from apoptosis in cerebral ischemia or OGD models [31, 32]. In contrast, Wang et al. reported that pretreated with 3-MA exerted detrimental neurological effects in a mouse MCAO model [33]. Our results showed that LC3 was overexpressed and that the expression level of p62 was strongly downregulated in the cortical tissue of ischemia.

Magnoflorine has been found to have antioxidant activity and to regulate autophagy [20, 27, 34]. It has been found to scavenge a stable free 2, 2-diphenyl-2-picryl-hydrazyl (DPPH) radical and to be capable of scavenging around 70.8% of all the free radicals at a dose of 50 *μ*g/ml [27]. In this study, we found that magnoflorine at both 10 mg/kg and 20 mg/kg could decrease the levels of NO and MDA. In addition, concentrations of GSH and GSH-PX were significantly increased only with the dose of 20 mg/kg. Furthermore, magnoflorine has been reported to promote the role of doxorubicin-mediated autophagy by upregulation of LC3-II expression. In addition, co-treatment with wortmannin, a blocker of autophagosome formation, markedly decreases doxorubicin/magnoflorine-induced LC3 conversion in breast cancer cells [34]. With these observations, we showed that magnoflorine treatment significantly decreased the fluorescence intensity of LC3 and the ratio of LC3-II to LC3-I in the ischemic brain tissues. The increment of p62 was also observed in the magnoflorine-treated group. These results indicate that magnoflorine has a neuroprotective effect through the regulation of oxidative stress and autophagy activation in a rat MCAO model. AMPK, as a cellular energy detector, has been proven to play a crucial role in the prevention of neuron injuries because of its activation influence for Sirt1 [17, 35]. Studies have shown that the activation of the AMPK/Sirt1 pathway protects against MCAO-induced impairments, which benefits cognitive function and neuron viability [35]. Our data showed that magnoflorine administration contributed to the activation of the Sirt1/AMPK pathway under cerebral ischemic conditions. The current study had a limitation. There are six rats in each group, which might have caused bias. Thus, future research needs to use larger rat samples at the individual level.

## 5. Conclusion

The results of the current study showed that magnoflorine treatment diminished MCAO-induced neurological damage in rats, which was potentially related to the suppression of oxidative stress and autophagy and the activation of the Sirt1/AMPK pathway. Our results suggest a promising benefit of magnoflorine therapy against neuronal injury in patients who suffered an ischemic stroke. However, further investigations aimed at defining the mechanisms of magnoflorine on cerebral ischemia are required.

## Data Availability

The datasets in this study can be obtained from the corresponding author upon reasonable request.

## Supplementary Materials

Supplementary Materials Supplementary materials: raw data of western blot.
