# Supplementary figures and images for: Magnoflorine Attenuates Cerebral Ischemia-Induced Neuronal Injury via Autophagy/Sirt1/AMPK Signaling Pathway
Source: Evid Based Complement Alternat Med. 2022 Sep 10;2022:2131561. doi: 10.1155/2022/2131561 (PMC9482485; doi:10.1155/2022/2131561)

## Supplementary materials

The raw data of WB are as follows:

**Fig. 4C**

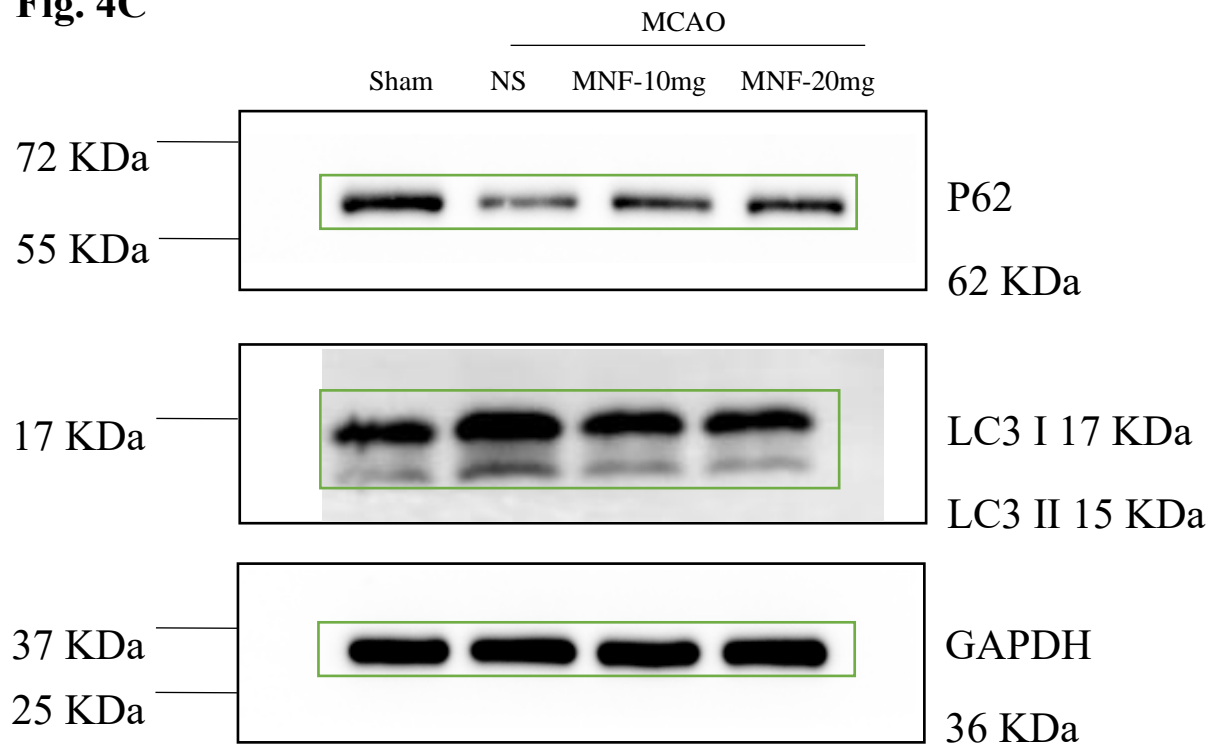

**Fig. 4F**

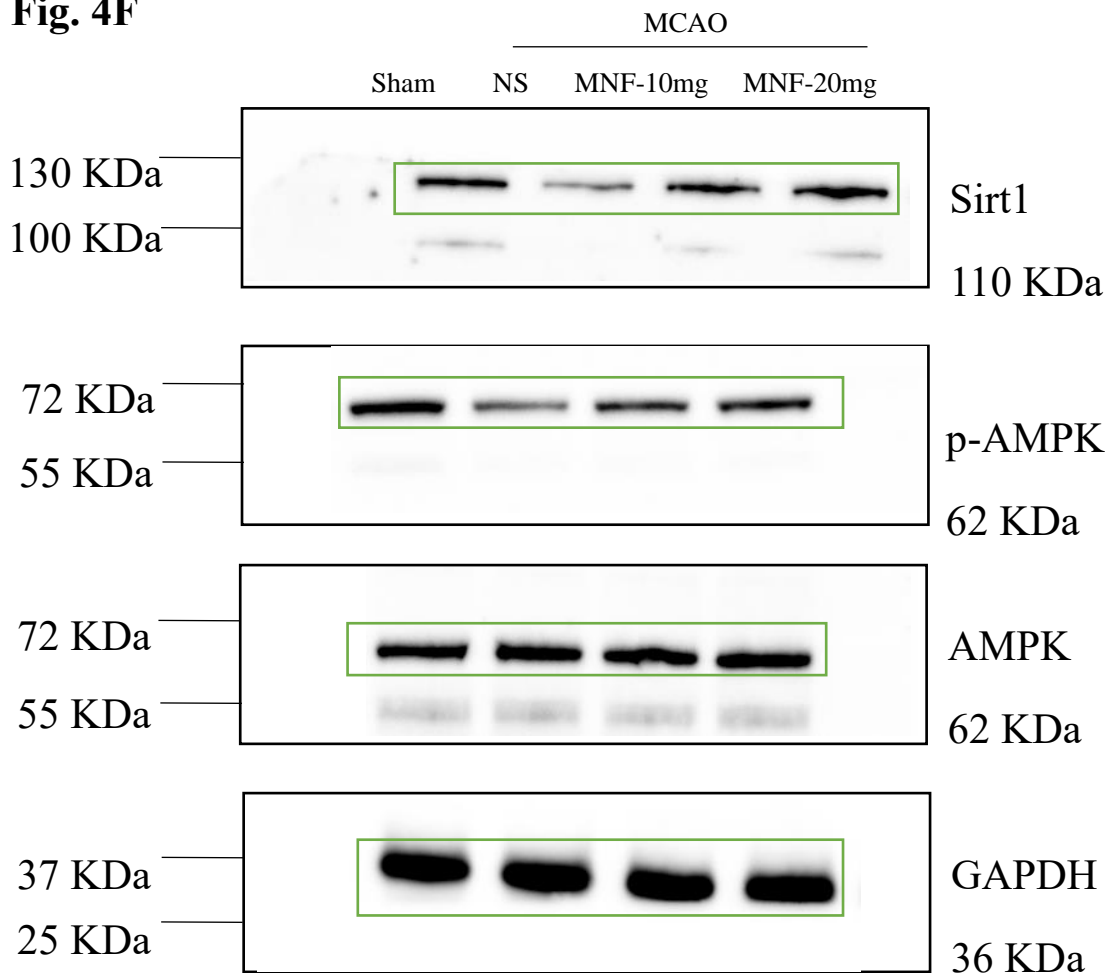

Supplement: Supplementary Materials — raw data of western blot. [file 2131561.f1.pdf]
